# Asymptomatic hepatic pseudoaneurysm identified 25 days after knife injury: a case report

**DOI:** 10.1186/s40792-023-01704-w

**Published:** 2023-06-27

**Authors:** Tomoyuki Sugi, Tsunehiko Maruyama, Reiji Nozaki, Masato Kawahara, Megumi Kamoshida, Sakura Narita, Hideya Takaku, Kazuaki Azuma, Yoshiro Chiba, Tatsuya Oda

**Affiliations:** 1 Department of Surgery, Mito Saiseikai General Hospital, 3-3-10 Futabadai, Mito, Ibaraki 311-4198 Japan; 2 Department of Gastrointestinal and Hepato-Biliary-Pancreatic Surgery, Faculty of Medicine, University of Tsukuba, 1-1-1 Tennodai, Tsukuba, Ibaraki 305-8575 Japan; 3 Department of Surgery, Kureha General Hospital, 1-1 Ochiai, Nishiki-Cho, Iwaki, Fukushima 974-8232 Japan; 4 Department of Cardiovascular Medicine, Mito Saiseikai General Hospital, 3-3-10 Futabadai, Mito, Ibaraki 311-4198 Japan

**Keywords:** Hepatic pseudoaneurysm, Liver trauma, Penetrating

## Abstract

**Background:**

Hepatic pseudoaneurysm (HPA) is a rare complication that can occur after liver trauma and carries a high risk of rupture. HPA is usually asymptomatic until rupture, so performing routine surveillance of liver trauma patients is important. Most posttraumatic HPA occurs within the first week after injury, so surveillance imaging ~ 7 days postinjury is suggested.

**Case presentation:**

We herein report a 47-year-old man who was diagnosed with asymptomatic HPA 25 days after a knife injury. The patient was transferred to the emergency room after attempting suicide by stabbing himself in his abdomen with a knife. The knife was surgically removed, and the postoperative course was uneventful. Computed tomography (CT) on postoperative day (POD) 12 showed no HPA. However, follow-up CT on POD 25 revealed HPA. The HPA was treated with coil embolization. The patient was discharged with no complications. One year after the injury, the patient had no recurrence or medical problems.

**Conclusion:**

When managing patients with penetrating liver trauma, it is important to note that HPA may not be identifiable on CT early after injury but may still develop later.

## Background

Posttraumatic hepatic pseudoaneurysm (HPA) is a rare complication that can occur after liver trauma, with an incidence of 2.9% [1]. HPA has a high risk of rupture and a high mortality rate, and hemorrhagic shock from HPA rupture has been associated with a 50% mortality [1].

HPA is often asymptomatic until it ruptures, so its early detection and treatment are important [2]. To avoid life-threatening hemorrhaging, routine surveillance is suggested five to seven days after liver trauma, as HPA is usually detected ~ 1 week after the injury on average [1, 2].

We herein report a case in which HPA was found on a postoperative day (POD) 25, despite computed tomography (CT) on POD 12 showing no HPA.

## Case presentation

**Fig. 1:**
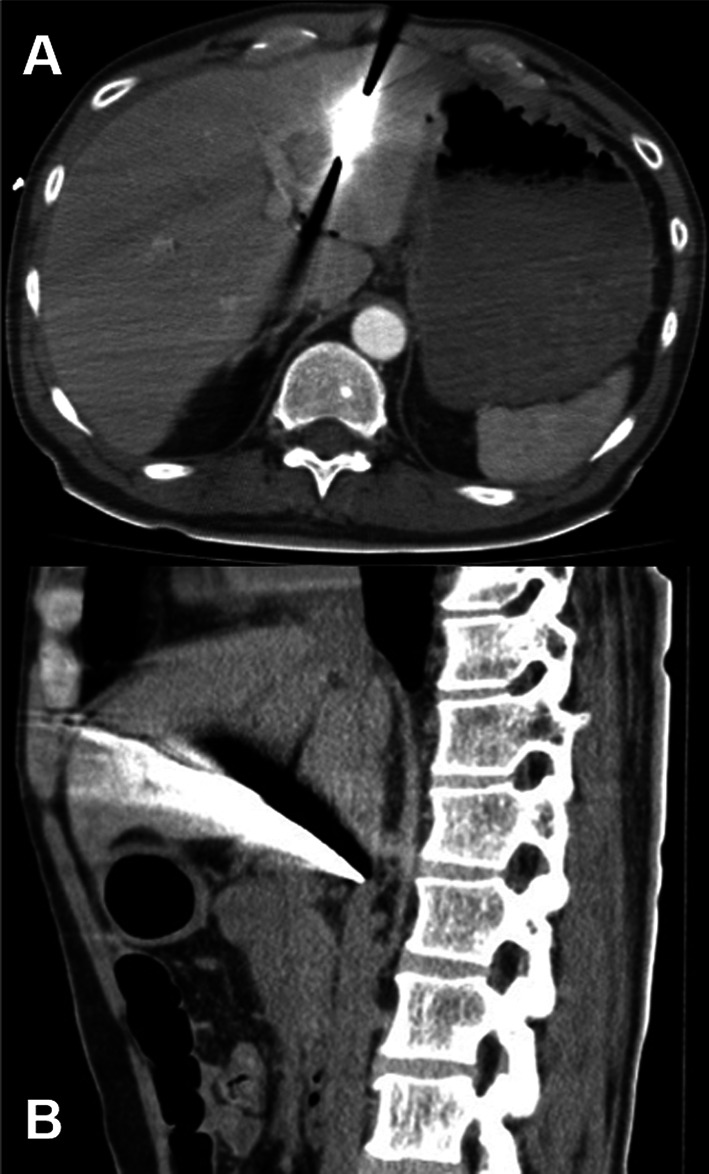
A knife penetrated the lateral segment of the liver, with the tip reaching near the inferior vena cava. **A** axial, **B** sagittal

A 47-year-old man was transferred to our emergency department 9 h after attempting suicide by stabbing himself in the abdomen with a knife. His medical history was unremarkable, and he had not previously received psychiatric care. He had a 3 cm puncture wound in his upper abdomen, and his omentum had prolapsed. His blood pressure and pulse rate were 76/58 mmHg and 117 beats/min, respectively. Focused assessment with sonography for trauma was negative. Tracheal intubation was performed, and the infusion raised his systolic blood pressure to 120 mmHg. After his vital signs stabilized, CT was performed. CT showed that a knife had penetrated the lateral segment of the liver, with the tip reaching near the inferior vena cava (IVC) (Fig. 1). There was no active extravasation or HPA. His blood test showed a white blood cell count of 21,000/μL, hemoglobin level of 12.4 g/dL, total bilirubin level of 0.34 mg/dL, alanine aminotransferase level of 28 U/L, and aspartate aminotransferase level of 75 U/L. The diagnosis was an abdominal stab wound with part of the knife remaining in the body. The possibility of IVC injury could not be ruled out.

**Fig. 2:**
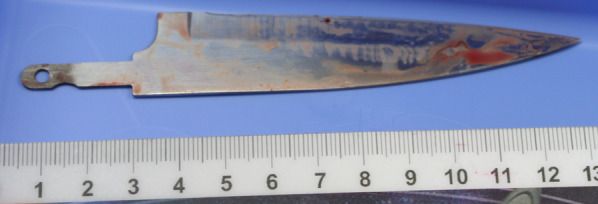
The knife with a missing handle was removed from the patient

**Fig. 3:**
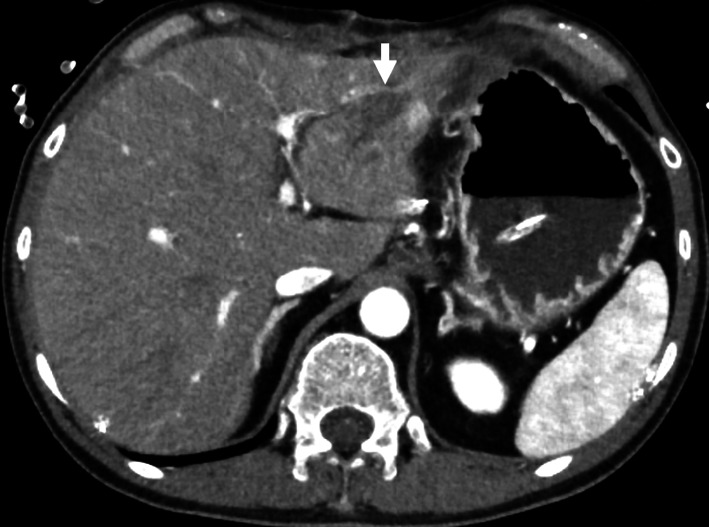
Computed tomography on postoperative day 12 showed a small amount of intrahepatic fluid in a penetrating wound in the lateral segment of the liver (the white arrow). There was no hepatic pseudoaneurysm

Emergency surgery was performed immediately. A cardiovascular surgeon also participated in the surgery in case the IVC was damaged. Penetrating wounds were observed in the lateral segment of the liver and the upper gastric body. The knife, with a missing handle, had penetrated the lateral segment of the liver and reached the hepatoduodenal ligament; it was removed manually (Fig. 2). There were no large vascular injuries, including injury to the IVC. Penetrating gastric wounds were closed using the Albert–Lembert technique with 3–0 absorbable braid and 3-0 nonabsorbable braid threads. Penetrating liver wounds were sutured using single nodal sutures with 1-0 absorbable braid threads. Drainage tubes were placed under the liver, under the right diaphragm, and in the pelvic cavity. Bleeding was controlled, and transcatheter arterial embolization (TAE) was not performed after surgery. Bile leakage from under the liver drainage tube was observed on POD 0. On POD 3, the pelvic cavity drainage tube also showed bile, and the patient developed lower abdominal pain. Therefore, the patient underwent reoperation for a bile leak on POD 3, as bile leaked from the penetrating wound in the lateral segment of the liver. The penetrating liver wounds were sutured using single nodal sutures with 1-0 absorbable braid threads. No bile leakage from the drainage tubes was observed after reoperation. CT on POD 12 showed a small amount of fluid in a penetrating wound in the lateral segment of the liver (Fig. 3). Residual bile leakage was suspected, but it remained intrahepatic, and conservative treatment was continued. There was no HPA (Fig. 3).

**Fig. 4:**
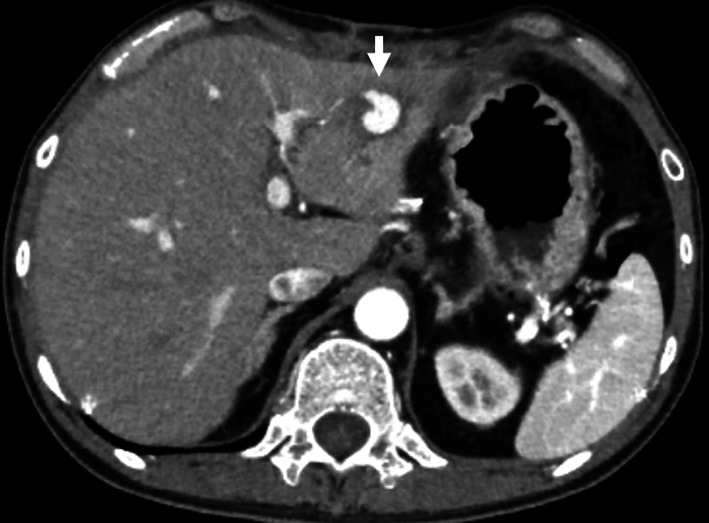
Computed tomography on postoperative day 25 revealed a hepatic pseudoaneurysm in the lateral segment of the liver. The white arrow indicates the hepatic pseudoaneurysm

**Fig. 5:**
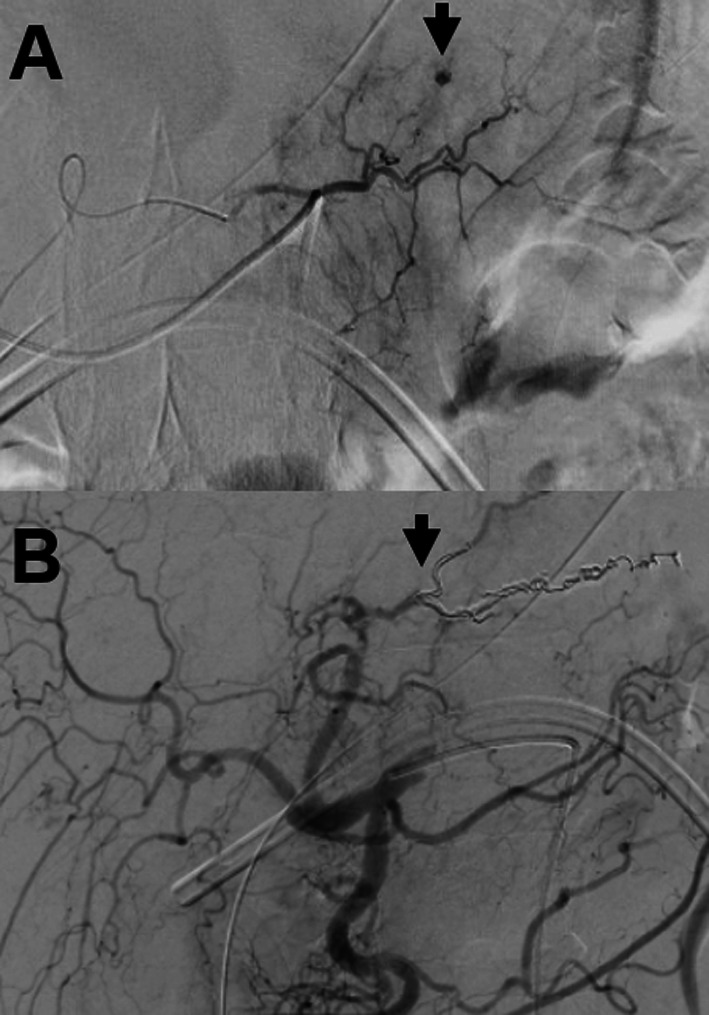
**A** The black arrow indicates hepatic pseudoaneurysm occurring in a branch of the left hepatic artery. **B** The black arrow indicates the coil embolization of the hepatic pseudoaneurysm

The patient had a persistent fever in the 37 °C range, and CT was performed on POD 25 to search for the source of the fever. CT revealed that the fluid within the lateral segment of the liver was decreased and there was no cause of fever, such as surgical site infection or an intra-abdominal abscess, but it did show HPA in the lateral segment of the liver (Fig. 4). On POD 32, the patient underwent coil embolization of the HPA that had developed in the branch of the left hepatic artery (Fig. 5). He was discharged on POD 40 with no complications. One year after embolization, the patient had no HPA recurrence or medical problems.

## Discussion

HPA is a false aneurysm that develops when an injured artery leaks into the surrounding tissue and can be caused by trauma, organ transplantations, infection, tumor, and surgery [3, 4]. Wagner et al. reported that 9% of patients with liver trauma developed complications, and 2.9% developed HPA [1]. The number of HPA patients with penetrating trauma is lower than that of HPA patients with blunt trauma because more patients are injured by blunt trauma than by penetrating trauma [1]. In proportion, the incidence of penetrating injury in HPA was shown to be higher than that of blunt trauma (penetrating: 4.9%, 7 of 143 patients; blunt: 2.2%, 11 of 491 patients) [1]. HPA has a high risk of spontaneous rupture, with reported mortality rates of 25% and 70% [5, 6].

As pseudoaneurysms are often asymptomatic until they rupture, their early detection and treatment are important [1]. Although angiography is the most valuable investigation modality for the diagnosis of HPA, for which it has 100% sensitivity [7], the incidence of HPA after liver trauma is only 2.9% [1]. Thus, it is not advisable to routinely perform angiography, which is an invasive procedure, in all patients. Therefore, follow-up CT plays an important role in detecting HPA in liver trauma patients before rupture [2]. By identifying HPA early, before it ruptures, patients and clinicians can avoid considerable morbidity and mortality as well as increased medical costs [1].

Regarding when to perform CT after an injury, Wagener et al. reviewed 634 hepatic trauma patients and suggested surveillance with interval CT 5–7 days postinjury, as the median time to HPA identification is 6.5 days [1]. In addition, Osterballe et al. also reported that the median follow-up period was five days [2]. Taken together, these previous reports suggest performing CT five to seven days after injury for HPA screening. However, in our case, despite no HPA being noted on CT performed at POD 12, it still appeared at POD 25. Therefore, HPA likely developed between POD 13 and 25 in our case. The possibility that bile leakage was involved in the development of delayed HPA cannot be ruled out. The patient might have developed a life-threatening condition due to HPA rupture if CT had not been reperformed. It is important to note that, in some cases, HPA can occur later, even if it is not detected on CT early after injury, as was the case for our patient.

Once HPA is diagnosed, regardless of its size, the risk of aneurysm rupture and acute hemodynamic compromise requires appropriate treatment [8]. Endovascular treatment including coil embolization, glue embolization, and angiographic stent placement, is the most common technique in hemodynamically stable patients [3, 9]. Some patients with hemodynamic compromise have been treated with endovascular treatment after perihepatic gauze packing [3, 10, 11]. Patients who are asymptomatic and hemodynamically stable, such as our patient, should receive endovascular treatment. Regarding our patient, coil embolization should have been performed earlier than seven days after identification, given the risk of rupture of the HPA.

We conducted a literature search using the keywords “hepatic pseudoaneurysm” and “penetrating” in PubMed (no time limit) and found four cases of HPA caused by penetrating trauma written in English, in addition to our case, that were included in our evaluation (Table 1) [3, 10, 11]. All five patients were men. The mean age of the patients was 25.4 years old, and the cause of injury was a gunshot in 3 patients, a knife in 1 patient, and a bomb blast in 1 patient. Two patients were asymptomatic, while the other three had symptoms that included abdominal pain, hematemesis, and light-headedness. According to the American Association for the Surgery of Trauma liver injury grade [12], the grade of hepatic injury was III in two patients, IV in one patient, and unavailable in two patients. The location of the pseudoaneurysm was the left lobe in two patients and the right lobe in three patients. Two of the 5 patients had bile leakage. All patients were diagnosed by CT. The mean duration from injury to HPA identification was 10.6 (3–25) days. All patients were treated with coil embolization. All patients were survived.

**Table 1:** Patients with hepatic pseudoaneurysm caused by penetrating trauma

Author	Age (years)/sex	Cause of injury	Symptoms	Grade of hepatic injury	Location of HPA	Bile leakage	Diagnosis method	Detected day from injury	Treatment	Prognosis
Ahmed [3]	23/M	Gunshot	Abdominal pain Light-headedness	IV	Right lobe	No	CT	14 days	Coil embolization	Survival
AbuAleid [10]	19/M	Bomb blast	Asymptomatic	Unavailable	Left lobe	No	CT	3 days	Coil embolization	Survival
AbuAleid [10]	25/M	Gunshot	Abdominal pain	III	Right lobe	No	CT	10 days	Coil embolization	Survival
Sidhu [11]	13/M	Gunshot	Hematemesis	Unavailable	Right lobe	Yes	CT	8 days	Coil embolization	Survival
Our case	47/M	Knife	Asymptomatic	III	Left lobe	Yes	CT	25 days	Coil embolization	Survival

*HPA* hepatic pseudoaneurysm, *CT* computed tomography, *TAE* transcatheter arterial embolization

Three of the five reported cases of HPA occurring after penetrating trauma, including the present case, were identified on days 10, 14, and 25 [3, 10]. This is despite previous suggestions to perform follow-up CT within 5–7 days postinjury [1, 2]. The reason for the late appearance of HPA after penetrating trauma may be explained in the report by Sandblom et al. [13]. They stated that in HPA after trauma, the injured artery is temporarily stanched by the pressure of a hematoma in the liver, but the hematoma is dissolved by bile from the damaged liver parenchyma, which eventually forms an HPA [13]. They also reported that in their experiments on dogs, liver wounds open to the peritoneal cavity healed earlier than those open to the gallbladder [13]. Based on these considerations, it is possible that penetrating liver trauma may maintain hematoma pressure on the injured artery longer because the hematoma is maintained longer. Accordingly, it takes the HPA longer to appear.

Patients with penetrating liver trauma have a higher incidence of HPA and may develop HPA later in comparison to patients with blunt trauma. Clinicians should be aware that some cases of HPA occur even beyond the 7 day period previously reported as ‘typical’.

## Conclusions

In the management of patients with penetrating liver trauma, clinicians need to be aware of the potential for HPA to occur later, even if initial surveillance imaging conducted shortly after the injury does not reveal HPA. This is due to the possibility that HPA may occur later after penetrating trauma than after blunt trauma, as has been observed in some previously reported cases.

## Data Availability

No additional data.

## Abbreviations

HPA: Hepatic pseudoaneurysm
CT: Computed tomography
IVC: Inferior vena cava
POD: Postoperative day
